# Sources of epigenetic variation and their applications in natural populations

**DOI:** 10.1111/eva.12946

**Published:** 2020-03-18

**Authors:** Bernard Angers, Maëva Perez, Tatiana Menicucci, Christelle Leung

**Affiliations:** ^1^ Department of biological sciences Université de Montréal Montreal Quebec Canada; ^2^ CEFE CNRS Université de Montpellier Université Paul Valéry Montpellier 3 EPHE Montpellier France

**Keywords:** bet‐hedging, developmental stability, epigenetics, holobiont, plasticity

## Abstract

Epigenetic processes manage gene expression and products in a real‐time manner, allowing a single genome to display different phenotypes. In this paper, we discussed the relevance of assessing the different sources of epigenetic variation in natural populations. For a given genotype, the epigenetic variation could be environmentally induced or occur randomly. Strategies developed by organisms to face environmental fluctuations such as phenotypic plasticity and diversified bet‐hedging rely, respectively, on these different sources. Random variation can also represent a proxy of developmental stability and can be used to assess how organisms deal with stressful environmental conditions. We then proposed the microbiome as an extension of the epigenotype of the host to assess the factors determining the establishment of the community of microorganisms. Finally, we discussed these perspectives in the applied context of conservation.

## 1 INTRODUCTION

**FIGURE 1:**
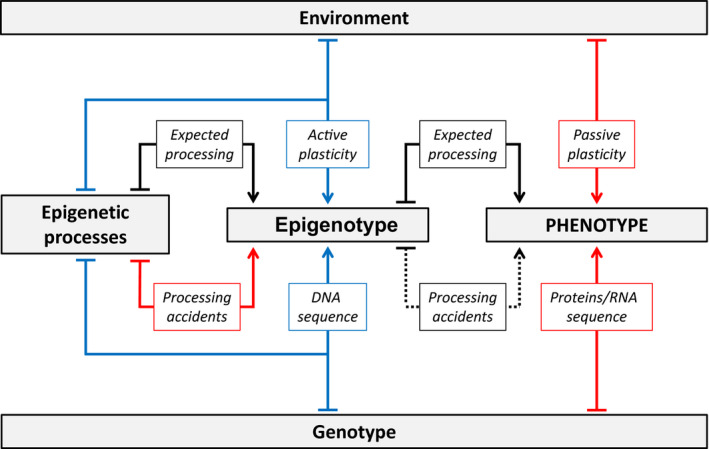
Sources of epigenetic and phenotypic variation. The formation of any phenotype resulted from a series of processes starting with gene expression. In a given environment, proteins and RNAs encoded by the genotype are expressed through a specific epigenotype. However, the realized phenotype can be different from the genetic expectations because of alterations by the environment (phenotypic plasticity). Red lines refer to the pure “environmental” (passive plasticity) and “genotypic” components. The epigenotype can be also modified by the interactions (blue lines) between epigenetic processes and genotype (obligated and facilitated epimutations) or “epigenetic processes and environment” (active plasticity), resulting in modifications of the “phenotype.” Finally, processing accidents can modify the epigenotype (pure “epigenetic” component) or the phenotype (developmental accidents). Processing accidents are stochastic (unpredictable results), but their rate of occurrence may be affected by genotype and environment. Epigenetic processes included the different components encoded by the genome and are therefore submitted to genetic and environmental factors

Phenotypic variation is central in ecology and evolution (Agrawal, 2001; Pigliucci, Murren, & Schlichting, 2006; Price, Qvarnström, Irwin, Qvarnstrom, & Irwin, 2003; West‐Eberhard, 1989). The phenotype is the target of natural selection as it determines the fitness of individuals in a given set of environmental conditions. Phenotypic variation is also the major key driver for individuals dispersal in heterogeneous environments and population persistence through environmental changes (Clobert, Galliard, Cote, Meylan, & Massot, 2009; Fitzpatrick, 2012; Forsman & Wennersten, 2016). The phenotype of individual represents the integration of the different components encoded by the genotype in a given environmental and epigenetic context (Peaston & Whitelaw, 2006). In addition to the effects of “pure” genetic and environmental components affecting directly the phenotype, epigenetic processes can, by themselves or in interaction with genetic or environmental factors, increase the number of options to modify the phenotype via the epigenotype (Figure 1).

Genetic variation is the cornerstone of intraspecific phenotypic variation. Alterations of the DNA sequence by mutations can modify the properties of proteins and noncoding RNAs as well as the level of gene expression and translation (Orr, 2005). Moreover, DNA is the particle of heredity, so the effects of evolutionary forces are recorded within a given population through generations. This genetic variation represents the reservoir of evolutionary opportunities.

Environment as the set of biotic and abiotic conditions can alter the phenotypic realization of a given genotype by affecting the development of the individual. Phenotypic plasticity refers to the property of one genotype to display different phenotypes according to environmental conditions (Pigliucci et al., 2006). Mechanistically, phenotypic plasticity could result from passive or active processes (Scheiner, 2006), and both can affect the same traits (Bell & Galloway, 2007). Passive phenotypic plasticity corresponds to phenotypic change not regulated by the organism, and resulting from changes in physicochemical conditions (e.g., temperature, salinity, nutrient availability) that alter properties of chemical, enzymatic, and cellular components (sensu* stricto* Whitman & Agrawal, 2009). Passive plasticity is then the phenotypic variation exclusively explained by the environment (Figure 1).

In contrast, the active phenotypic plasticity (hereafter referred as phenotypic plasticity) is characterized by phenotypic changes resulting from modifications of gene expression and developmental pathways induced by the environmental conditions (Scheiner, 2006; Whitman & Agrawal, 2009). Epigenetics is the underlying mechanism responsible for phenotypic plasticity, in interaction with the environment (Figure 1) and the central theme of this special issue perspective (Boxes 1, 2, 3). Different environmental signals perceived during development are reflected in specific epigenotypes (Kucharski, Maleszka, Foret, & Maleszka, 2008). This property is exploited by several organisms to produce distinct phenotypes without genetic determinism such as sex determination in some reptile and fish species (Navarro‐Martín et al., 2011; Valenzuela & Lance, 2004) or cast differentiation in honeybees (Kucharski et al., 2008).

Box 1 **Personal thanks from one of the authors to Dr B.**

Dear Dr B.

Because all my previous attempts sounded like texts that end up in obituary columns, I rather decided to write you a letter. However, I was not sure who to send this letter to. I could have written to my thesis co‐supervisor. I would have thanked you for having believed in my weird ideas and for guiding me to realize them. I would have told you how my research interests in ecology and evolution have been heavily skewed towards a molecular perspective thanks to your contagious enthusiasm; that these years I spent in your lab to do genetic landscape before the discipline bears this name have not only been decisive for my career but was also one of the best moments of my life. I could also have written a letter to the mentor that I have always admired. I would have explained to you how the “LB”, varying from zero to one, is my unit of measure for career achievement in research. But what I really want is to write to a friend. A friend I do not often see, for many reasons, but that I always appreciate. Thank you.

Bernard Angers

Box 2 **A review of our research program in epigenetic of natural populations**

B. Angers’ laboratory focusses on variation in DNA methylation in natural populations, especially on clonal vertebrates. Both fish *Chrosomus eos‐neogaeus* (top) and salamander *Ambystoma laterale‐jeffersonianum* (bottom) reproduce asexually, through gynogenesis and kleptogenesis, respectively. The rational of using asexual vertebrate models is to have biological replicates without genetic variation. In our first review in Molecular Ecology (Angers, Castonguay, & Massicotte, 2010), we highlighted the relevance of epigenetic processes as real‐time mechanisms allowing survival of organisms through environmental fluctuations, a *sine qua none* condition for persistence and evolution. Our pioneering studies in natural populations of a nonmodel vertebrate revealed higher levels of DNA methylation variation compared with mutations (Massicotte, Whitelaw, & Angers, 2011) and that epigenetic variation is environment‐specific (Leung, Breton, & Angers, 2016; Massicotte & Angers, 2012; Massicotte et al., 2011). Epigenetic marks can be randomly established and environmentally induced (Massicotte & Angers, 2012), and their relative abundance is correlated to the predictability of environmental changes (Leung et al., 2016), as expected from plasticity and diversified bet‐hedging. The particular reproduction mode of these models also allows individuals to incorporate locally adapted genome within a hybrid genotype. Such unusual genome rearrangement enables to disentangling the relative importance between adaptation and plasticity (Beauregard & Angers, 2018). Finally, we also used DNA methylation pattern as a molecular tool to assess environmental conditions (Angers, Dallaire, Vervaet, Vallières, & Angers, 2012; Angers et al., 2018) or physiological conditions of organisms (Leung, Angers, & Bergeron, 2020). This article reflects the current perspectives addressed in our laboratory.
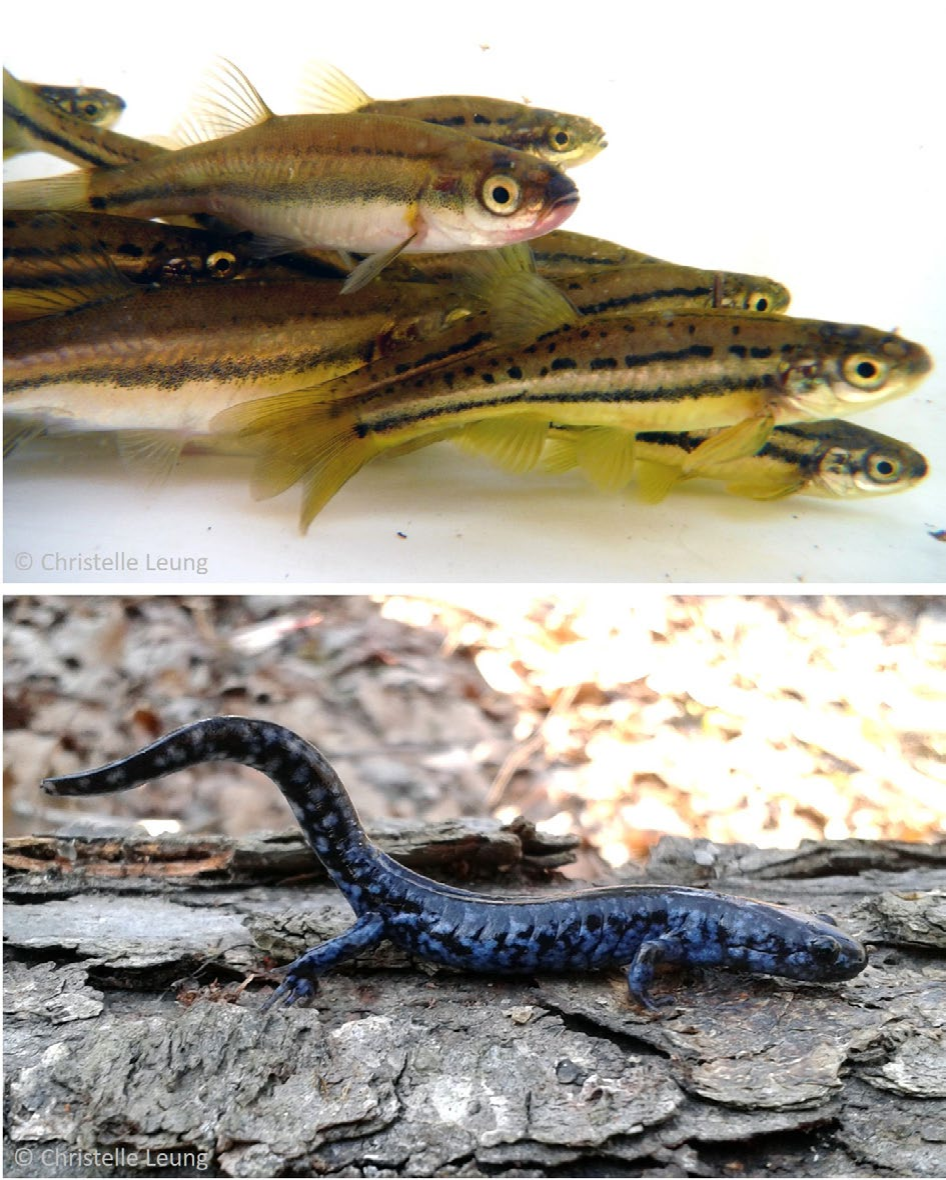

Box 3 **Epigenetics**

A mechanistic definition of epigenetics is a set of real‐time fine‐tuning mechanisms allowing the cell(s) of an organism to respond to intrinsic and/or extrinsic environmental conditions. The large diversity of epigenetic processes encompasses histone modifications (e.g., acetylation), DNA modifications (e.g., cytosine methylation), and non‐coding RNAs (Duncan, Gluckman, & Dearden, 2014). These mechanisms are grouped together as processes regulating gene products in terms of pretranscriptional or post‐translational processes (Murrell, Rakyan, & Beck, 2005). They control gene pathways by modifying the chromatin or the genetic landscapes via the epigenotype. The epigenotype refers to the chemical tags established by the epigenetic machinery, such as profiles of DNA methylation and histone proteins.

Several sources can modify the epigenotype (Box 4). These modifications are extremely dynamic and reversible, making them the proper hallmarks of epigenetics. For instance, environmental stress can alter DNA methylation within minutes interval and reset after 48 hr (Huang et al., 2017). Most of the active epigenetic marks on the genome are not expected to last longer than one generation (Horsthemke, 2018; Kazachenka et al., 2018). Even in processes that required transgenerational inheritance of epigenetic marks such as genomic imprinting in mammals, these marks are reset during gametogenesis (Miyoshi et al., 2016). This makes epigenetics a fundamental mechanism to produce different phenotypes without genetic determination.

Epigenetic components (such as DNA methyltransferases) are encoded by the genome, and their transmission depends on how they adequately adjusted the phenotype according to the environment. The epigenetic machinery is then an evolving trait responding to selection, a phenotype‐specific to each organism according to its evolutionary history. This explains the wide variation existing around epigenetic processes among organisms, well exemplified by the difference between plants and animals (Feng et al., 2010). For instance, the transgenerational inheritance commonly observed in plants whereas this phenomenon is anecdotic in animals (Horsthemke, 2018) illustrates how such properties could be taxon‐specific.

Finally, in a given environmental condition, processing errors (Figure 1) during the development are expected to increase variance around the target phenotype. A large portion of the phenotypic variation is therefore explained neither by genetic variation nor by environmental conditions (while both environment and genome factors may affect the rate at which such errors occur (Leary & Allendorf, 1989; Parsons, 1992)). This could be easily observable in clonal organisms reared in a given environment (Leung, Breton, & Angers, 2016, 2018).

Among these processing errors, “pure” epigenetic changes result from errors in the establishment and the maintenance of the epigenotype (Figure 1). They refer to the stochastic variation that is independent of environmental conditions and the genetic background (Horsthemke, 2006; Lorincz, Schubeler, Hutchinson, Dickerson, & Groudine, 2002). Because of the fundamental role of epigenetic processes in regulating gene expression, errors in the epigenotype can result in phenotypic variation (Cubas, Vincent, & Coen, 1999). Stochastic epigenetic changes occur several orders of magnitude higher than mutations (Bennett‐Baker, Wilkowski, & Burke, 2003; Massicotte & Angers, 2012; Schmitz et al., 2011) and are therefore an important source of phenotypic variation, adaptive or not.

If the discovery of molecular hallmarks of epigenetic processes is relatively recent (and still in progress), several effects of epigenetics in natural populations have been indirectly studied for several decades by evolutionary biologists and ecologists through quantitative genetics, development, plasticity, and bet‐hedging. Once controlled for genetics and environment, phenotypic variation is expected to be a good proxy of epigenetic variation. However, the phenotype only reveals a small portion of the epigenetic variation and does not provide information on the epigenetic sources responsible for the phenotypic variation.

Epigenetics is intimately associated with the mechanisms orchestrating the developmental pathways of organisms in a given environment. The epigenotype is then expected to include valuable molecular markers to assess the environmental conditions experienced by individuals. Development of several methodological approaches relevant in ecology and evolution (reviewed in Rey et al., 2019) allows extensive analysis of the epigenotype, the molecular signature underlying epigenetic processes, and gives access to hundreds of thousands of molecular markers. In this paper, we then discussed how assessing the sources of epigenetic variation provides useful predictions and conceptual frameworks in three different topics in ecology and evolution.

### 1.1 Strategies to face environmental fluctuations

One of the fundamental properties of organisms is to rapidly adjust their phenotype without relying on genetic variation. For instance, ecological strategies such as phenotypic plasticity and diversified bet‐hedging rely on different sources of epigenetic variation (Box 4) to face environmental fluctuations. These strategies are expected to display specific pattern of epigenetic variation that could be detected and quantified through molecular and statistical approaches. Disentangling the different factors responsible for the production of a phenotype (Figure 1) is a *sine qua non* condition to assess how organisms evolved to face environmental changes.

Box 4 **Sources of epigenetic variation**

Variation at the level of the epigenotype can be partitioned in three different sources: changes resulting from (a) the interaction between environment and epigenetic processes (environmentally induced), (b) the interaction between genotype and epigenetic processes (genetically induced), (c) or epimutations, the “pure” epigenetic source of variation.

Environmentally induced epigenetic changes are triggered by intrinsic as well as extrinsic environmental conditions (Atlasi & Stunnenberg, 2017; Horvath, 2013). Epigenetic processes allow adjustment of gene expression according to environmental conditions, the underlying mechanism of phenotypic plasticity (Angers et al., 2010; Bollati & Baccarelli, 2010). Some epigenetic changes could be extremely dynamic (e.g., circadian cycle; Azzi et al., 2014; Coulson et al., 2018; Lim et al., 2017; Stevenson, 2017) according to their lability/flexibility during the development (Box 5). While epigenetic marks are mitotically transmitted, DNA methylation profiles are known to change through the life span of an individual and represent a reliable molecular estimator of biological age (epigenetic clock; Horvath, 2013).

Genetically induced changes are associated with specific mutation in obligate epigenetic variation or could be influenced by the genotype in facilitated epigenetic variation (Horsthemke, 2006; Richards, 2006). They are expected to represent a large proportion of epigenetic variation, between 22% and 80% of interindividual variability of DNA methylation in humans (Bell et al., 2011; Gertz et al., 2011; Greally, 2017). Such epigenetic marks could be confused with epigenetic heritability because the same epigenetic changes are more likely to occur than random epimutations (Horsthemke, 2018).

Epimutations refer to stochastic epigenetic changes resulting from processing accidents. They reflect the incapacity to adequately organizing or maintaining consistent epigenetic marks. These errors can occur at a higher rate than mutations (Bennett‐Baker et al., 2003; Massicotte et al., 2011; Schmitz et al., 2011). They could be useful to generate phenotypic variation in the context of diversified bet‐hedging strategies (Casadesús & Low, 2013; Herman, Spencer, Donohue, & Sultan, 2014; Piggot, 2010).

### 1.2 Epigenetic asymmetry

Departures from optimal developmental conditions are expected to increase developmental instability (Klingenberg, 2019; Møller & Swaddle, 1997; Rott, 2003). Stochastic epigenetic changes as consequence of the developmental instability can occur when an organism fails to buffer environmental disturbances, such as exposure to stressing environmental conditions during the development. These errors result in greater susceptibility of inaccuracies in the production of the phenotype (DeWitt, Sih, & Wilson, 1998; Dongen, 2006; Leung, Forbes, & Houle, 2000; Markow, 1995; Rott, 2003). If the organism is not able to develop in a precise path (canalization) or to buffer these perturbations (developmental stability), differences are going to accumulate through development. In bilaterally symmetrical organisms, left and right sides are expected to follow, to some extent, similar but independent developmental courses. However, despite having the same genome and being exposed to the same environment, the development of each of the body sides is subject to random processes and is expected to deviate from each other, leading to asymmetry (Klingenberg, 2003; Palmer, 1996). It has then been proposed that asymmetry resulting from such stochastic errors (fluctuating asymmetry) is a measure of developmental instability (Møller & Swaddle, 1997; Palmer, 1996; Palmer & Strobeck, 1986). Traditionally inferred through morphological analyses, we discussed how developmental instability could be assessed through epigenetic analyses.

### 1.3 The microbiome

In addition to the individual's developmental pathway, the phenotype can also be strongly influenced by its microbiome, the more or less complex and specific community of microorganisms (bacteria, archaea, fungi, protists) that an organism may host. On the one hand, the microbiome can be considered as another set of environmental conditions affecting the host. On the other hand, the concept of the holobiont was proposed to consider the complete system facing natural selection. In this context, the metagenome of the microbiome together with the genome of its host forms the genome of a multispecies individual. However, both concepts suffer from major limitations since on the one hand the composition of the host's microbiome is genetically and environmentally determined and on the other it is not strictly heritable. We proposed that the microbiome should be considered as an environmentally acquired component of the host epigenotype. The microbiome is then not just an additional set of genes or environmental conditions but could be considered as an extension of the flexible epigenotype of the host. This concept may be helpful in investigating the role of host genotype, environment, and stochasticity in the establishment of the microbiome and allow considering plasticity and diversified bet‐hedging strategies at the level of the microbiome.

We concluded this paper by integrating these perspectives in a conservation aspect, to illustrate the usefulness of epigenetic variation in an applied context.

## 2 STRATEGIES TO FACE ENVIRONMENTAL FLUCTUATIONS

Adaptation is an optimization process of the genetic variation toward a higher fitness in a given environmental condition, traduced by the increase in frequency in the population of sets of alleles allowing the best fitness. Specifically, adaptation to a given environmental condition generally requires it remains stable through time, so a population can explore the adaptive landscape (Svensson & Calsbeek, 2012). However, environmental conditions can fluctuate within and among generations, according to cyclic processes (e.g., circadian cycles, seasons), or stochastically (e.g., resource availability, variation around a mean temperature or precipitation). As a consequence, the production of a single phenotype resulting from a locally adapted genotype might not be the optimal option in a rapidly changing environment (Burger & Lynch, 1995; Lande & Shannon, 1996). For instance, the phenotype developed under some conditions would not necessarily be optimal for the whole lifespan of an individual, or environmental fluctuations among generations would require different phenotypes between parents and offspring (Gluckman, Hanson, & Spencer, 2005; Uller, 2008). Similarly, phenotypic variation is advantageous for genetically uniform populations (clonal organisms) to colonize heterogeneous environments (Vrijenhoek & Parker, 2009).

Two ecological strategies—phenotypic plasticity and diversified bet‐hedging—enable the development of distinct phenotypes from a single genotype to buffer environmental heterogeneity and fluctuation, and these strategies could be characterized according to the different sources of epigenetic variation they rely upon (Box 4).

### 2.1 Phenotypic plasticity

The development of plastic traits relies on a flexible gene pathway modulated via environmental cues (phenotypic plasticity—Scheiner, 1993, 2006; Stearns, 1989). The strong differentiation among cell types bearing the same genome in multicellular organisms is a spectacular example (Waddington, 1942, 1953, 1957), but this can also be extended to an individual's phenotype. Phenotypic plasticity is usually illustrated as a reaction norm, where the level of plasticity is assessed by phenotypic differences between environments (e.g., the regression slope of a linear reaction norm). Shapes and slopes of reaction norm generally differ among genotypes, suggesting a genetic basis of phenotypic plasticity (Gavrilets & Scheiner, 1993; Scheiner, 1993; Schlichting & Pigliucci, 1998; Stearns, 1989).

Theoretical works have demonstrated that plastic genotypes would be selected for dealing with predictable environmental changes (Botero, Weissing, Wright, & Rubenstein, 2015; DeWitt et al., 1998; Reed, Waples, Schindler, Hard, & Kinnison, 2010; Scheiner & Holt, 2012). A change is defined as predictable when the environment at the timing of phenotype's development is correlated to the environment of its selection, or unpredictable when there is no correlation between cues triggering the development of a given phenotype and its environment of selection (Scheiner, 2014b; Scheiner & Holt, 2012). Therefore, phenotypic plasticity is the process by which organisms can anticipate environmental conditions from a given signal and produce a phenotype expected to fit with such future environment (Leung et al., 2020; Scheiner, 1993). Because epigenetics is responsible for the fine‐tuning of gene expression (Box 3) and the environment represents a major source of epigenetic variation (Box 4), it has been proposed that environmentally induced epigenetic variation mediates phenotypic plasticity (Angers et al., 2010; Bollati & Baccarelli, 2010).

### 2.2 Diversified bet‐hedging

Individuals can also rely on phenotypic variation achieved by random processes (Box 4). The diversified bet‐hedging is a risk‐spreading strategy based on the capacity of increasing variation around a median state of a character. This strategy bets that at least one of the produced phenotypes would fit with the actual environmental conditions, allowing a non‐null fitness of the population in fluctuating environments (de Jong, Haccou, & Kuipers, 2011; Slatkin, 1974; Veening, Smits, & Kuipers, 2008).

Random production of variable phenotypes, irrespective of environmental conditions, ensures the persistence of populations coping with unpredictable environmental changes (Acar, Mettetal, & Oudenaarden, 2008; Balaban, Merrin, Chait, Kowalik, & Leibler, 2004; Botero et al., 2015; Kussell & Leibler, 2005; Scheiner, 2014a; Scheiner & Holt, 2012). Random establishment of epigenetic marks (Box 4) could be among the mechanisms underlying diversified bet‐hedging strategy (Casadesús & Low, 2013; Herman et al., 2014; Piggot, 2010; Vogt, 2015, 2017).

### 2.3 Epigenetic characterization of the ecological strategies

The adaptiveness of the different ecological strategies according to the predictability of changes has been demonstrated through theoretical works (Botero et al., 2015; Scheiner, 1993, 2013; Scheiner & Holt, 2012). But few empirical studies have tested these predictions, mostly because of measurement difficulties in natural environments: Long time series and multiple replicates are necessary to assess environmental predictability. Similarly, in the case of bet‐hedging, stochasticity in phenotypic variation could be difficult to assess in an individual‐centered design, as large sample size is needed; for nonclonal biological models, random phenotypic variation cannot be distinguished from genetic variation; and finally, phenotypic data collection should be performed before selection to avoid a confounding effect with environmental influence on phenotypic variation.

Quantitative genetics is a traditional approach that aims at linking phenotypic variation to underlying genotypes, by disentangling environment, genetics (heritability), and the interaction environment × genetics on phenotypic variation (Falconer, 1960). However, not all traits are quantitative and phenotypes could be difficult to record. For instance, comparisons of distinct species could be difficult when different traits are involved (no generalization is possible). Phenotype characterization according to the environment also requires the knowledge of the precise environmental cue responsible for the development of a trait, while labile traits could display variation within individuals with long generation intervals (Box 5). It might also be difficult to disentangle developmental and selection effects on phenotypic variation in natural populations, as the measured variation would already be the result of a previous selection on a higher developmental phenotypic variation. Finally, even the transcriptome that is a precise phenotypic measure to detect changes in gene expression does not provide information on how the epigenetic variation was established. As a consequence, the distinction between phenotypic plasticity and bet‐hedging strategies could be difficult if measured only by the phenotypic variation.

Box 5 **Flexibility of traits and developmental pathway**

The complexity of living organisms involves the diversity of genes and related traits, that are expressed during (and after) development, continuously or not. Traits that require stability during the life of an individual are expected to display a short period of epigenetic lability during the development (e.g., polyphenisms). At the opposite end of the spectrum, traits requiring changes later throughout the individual's life would be associated with loci displaying higher epigenetic instability or sensitivity toward environments (flexibility). By analogy to Waddington's epigenetic landscape representing embryonic development (Waddington, 1942, 1953, 1957), one can extend this concept to trait development. Different phenotypes could, therefore, be categorized by a gradient of flexibility in terms of epigenetic reprogramming, similar to cell differentiation, but where more flexible phenotype could display alternative states late in the development or during adulthood 
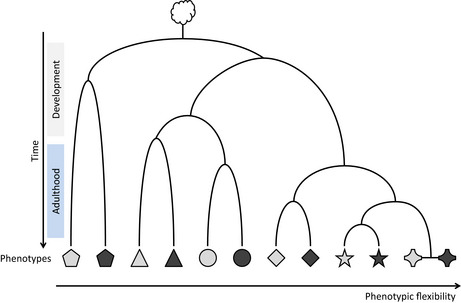
.

The figure shows the flexibility of traits according to their developmental pathway. At each node, the developmental faith of a given trait could be triggered by a specific environmental cue (phenotypic plasticity) or resulted from stochasticity (bet‐hedging). Only two possibilities are represented at each node to simplify the figure. Symbols represent different traits, while colors (gray versus black) represent the different states of a given trait.

Such a development‐related flexibility of epigenetic reprogramming should therefore be taken into account in the partitioning of the different sources of epigenetic variation. This is analogous to the covariation between shape and size throughout the development in ontogenetic allometry that provides useful tools to address specific questions concerning development (Shea, 1985).

Assessing epigenetic variation could help to better understand the inherent mechanisms of the different ecological strategies. Epigenetic variation is a good candidate for ensuring variation of traits (Cubas et al., 1999; Jaenisch & Bird, 2003; Kucharski et al., 2008; Manning et al., 2006; Suzuki & Bird, 2008). Thereby, epigenetic processes could underlie phenotypic flexibility, by being the first level of change in anticipation of future environmental conditions and ensuring the production of the fittest phenotype for each environmental change that could happen several times during the life of an organism (Gluckman, Hanson, & Low, 2011; Leung et al., 2020; Masri & Sassone‐Corsi, 2010). Different hypotheses can be formulated. For example, bet‐hedging strategies could rely on epigenetic changes that occur globally all over the genome. That could be the result of a high rate of methyltransferase errors resulting in higher stochastic DNA methylation patterns (Castonguay & Angers, 2012; Goyal, Reinhardt, & Jeltsch, 2006). Alternatively, low error rates and more accurate methyltransferase enzymes would reflect the propensity of a genotype to achieve a better canalized phenotypic development. In such a case, several loci/genes would consistently respond epigenetically to the same environmental change. Finally, another hypothesis would be that epigenetic changes are gene/locus‐specific. This would result in a group of genes behaving specifically in response to an environmental change. Integration of environmental signal by the genotype to display epigenetic changes and thus the production of an environmentally driven phenotype could be trait‐dependent (Box 5). Depicting the sources of phenotypic variation should not be limited to the analyses of DNA sequences but should also include environmental factors and a stochastic component. Thus, quantitative genetics is used to partition phenotypic variation in estimating the portion of heritability of a given phenotype compared with environmental and random effects. As an additional layer, we propose a quantitative analysis of epigenetic variation. Such analysis may allow assessing the different sources of phenotypic variations (Figure 1; Box 4), in addition to unraveling the capacity of a genotype to cope with changing environments. Partitioning phenotypic variation according to epigenetic variation was previously proposed by Banta and Richards (2018). Such an approach would be useful to identify genomic regions to given phenotypic variation. Instead, we suggest disentangling the different sources of epigenetic variation. Considering epigenetics as a character, a quantitative analysis of epigenetic variation, in this case, is a matter of quantitative genetics. But the main difference with classic quantitative genetics (i.e., the partition of the sources of trait variation) is the fact that using epigenetic variation instead of a given phenotype brings additional precision enabling to assess the ecological strategies used by organisms. For instance, part of the epigenetic variation could be neutral and reflect how the epigenetic marks have been established (i.e., randomly versus environmentally driven), whereas most of the traits have already been subject to selection at the moment of the measurement. Therefore, a quantitative analysis of epigenetic variation would help to assess the capacity of a genotype to display epigenetic variation according to either environmental conditions or stochastic processes.

Epigenetic variation represents a good candidate of the nongenetic molecular signature of the different ecological strategies. For instance, quantitative analysis of the clonal fish *Chrosomus eos‐neogaeus* DNA methylation is strongly influenced by the genome (10% of genetic effect on epigenetic variation (Leung et al., 2016)). Furthermore, it has also been shown that lineages sampled in predictable environments displayed a higher proportion of environmentally induced epigenetic variation compared with lineages from unpredictable environments (Leung et al., 2016). These empirical results were consistent with theoretical models predicting the selection of contrasting ecological strategies—that is, phenotypic plasticity (environmentally induced epigenetic variation) or bet‐hedging (stochastic epigenetic variation)—according to environmental predictability (Botero et al., 2015; De Jong, 1999; Gavrilets & Scheiner, 1993a; Tufto, 2015).

## 3 EPIGENETIC ASYMMETRY

Developmental instability is related to developmental perturbations, such as modifications in gene expression and other processing accidents (Figure 1), resulting in variation of the produced phenotype among individuals (Klingenberg, 2003; Scheiner, 2006). These perturbations can arise either stochastically (e.g., epimutations) or in response to stressful environmental conditions (Scheiner, 2006; Zakharov, Zhdanova, Kirik, & Shkil, 2001).

For instance, in the case of phenotypic plasticity (interaction environment × epigenetics; Figure 1), the basal pathway of a given trait is expected to be stable, since it is determined by coadapted gene complexes (Dobzhansky, 1951). Alternative developmental pathways could result in developmental instability: when the individual fails to buffer environmental disturbances, which could result in imprecise development (DeWitt et al., 1998; Dongen, 2006; Leung et al., 2000; Markow, 1995; Rott, 2003). This increase in developmental instability could be due to (a) the use of noncoadapted genes that may not collaborate so harmoniously (Clarke, 1993); (b) indirect effects on other traits (e.g.*,* pleiotropy, epistasy) (Pigliucci, 2005; Scheiner, 1993); and (c) unrelated structures being indirectly affected just for being epigenetically coupled to the actual target (i.e., due to physical proximity) (Clarke, 1993; Scheiner & Northern, 1991). Moreover, the environmental signal that triggered the alternative pathway of phenotypic plasticity can, per se*,* also increase developmental noise (Møller & Swaddle, 1997). The stochastic changes resulting from developmental instability accumulate throughout the development. During the development of symmetrical organisms, the body sides are then expected to deviate from each other which, subsequently, lead to asymmetry (Klingenberg, 2003; Palmer, 1996).

In bilateral organisms, different kinds of left–right asymmetries are characterized by the frequency distribution of their signed right‐minus‐left variation, with well‐defined statistical attributes (Palmer & Strobeck, 1986). Directional asymmetry, for instance, reflects a precise developmental bias toward one side of the body, either left or right (Levin, 2005; Palmer & Strobeck, 1992). The signal triggering the asymmetrical development can be genetic or environmental (Klingenberg, 2003; Leung, Duclos, Grünbaum, Cloutier, & Angers, 2017; Levin, 2005; Palmer & Strobeck, 1992). Therefore, the pattern is consistent within a given group but can vary among groups. A classic example is the lateralization of the body in flatfishes (order Heterosomata) following the migration of one eye from one side to the other. Flatfish species are consistently all dextral or all sinistral and specimens showing an asymmetry to the opposite side are considered abnormals (Hubbs, Hubbs, Hubbs, & Hubbs, 1945).

Fluctuating asymmetry, on the other hand, reflects the compromise between two independent and opposite developmental processes: developmental noise and developmental stability, and may arise either as a result of an increase in the former or a decrease in the latter (Palmer, 1994). Fluctuating asymmetry is therefore considered as one of the phenotypic outcomes of developmental instability (Klingenberg, 2003). In bilateral organisms, it is traditionally calculated from morphometric differences between left and right sides, where the signed right‐minus‐left variation has a parametric mean of zero and is normally distributed (Palmer, 1994). However, to properly detect fluctuating asymmetry differences among samples can be challenging and demands caution during both measurements and analyses (Palmer, 1994). Either low number of traits used, or moderate measurement errors, for example, can conceal and/or bias the estimation of truly existent departures from asymmetry. This limits the use of phenotype to estimate developmental instability, not to mention the dependence on trait size, the developmental dependence among distinct traits, and the variation due to individuals’ stage of development/age (Palmer, 1994).

As an alternative (or a complementary approach), we propose the use of epigenetic variation to assess asymmetry. The epigenotype is tissue‐specific; a given tissue from the different sides of a bilaterally symmetric individual is thus expected to show, to some extent, similar patterns. However, the opposite body sides are exposed to random developmental errors occurring locally. This result in differences that could be perceived between left and right sides when analyzing opposite structures or tissues of bilateral organisms. Hence, it would be possible to estimate developmental instability by measuring the variance of the left–right differences (DeWitt, 1998; DeWitt et al., 1998) of epigenetics marks in such structures, what we call the epigenetic fluctuating asymmetry.

We could then predict that, in the absence of genetic variation, the epigenetic fluctuating asymmetry would be higher on individuals presenting a less stable regulation of gene expression, such as (a) individuals raised in stressful conditions (i.e., increased noise), and/or (b) resulting from alternative developmental pathways (e.g.*,* by plasticity), reflecting the increased developmental instability.

We tested this hypothesis by comparing fluctuating asymmetry assessed from morphological and DNA methylation left–right variation in *Chrosomus eos‐neogaeus* clonal individuals. This fish shows a polyphenism with either symmetric or asymmetric dental formula on their 7th pharyngeal arch, characterizing a basal and alternative developmental pathways, respectively (Leung et al., 2017). As expected from the literature (Palmer, 1994; Palmer & Strobeck, 1986; Waddington, 1942), the alternative pathway is less precise and the individuals with asymmetric dentition showed higher levels of fluctuating asymmetry at the level of the shape of the arches as a trade‐off for exhibiting the plastic phenotype (Leung et al., 2017). Fluctuating asymmetry was assessed from differences between left and right sides of the body for both 3D geometric morphometric analysis of the pectoral appendage and DNA methylation variation of lateral muscle (Appendix S1).

**FIGURE 2:**
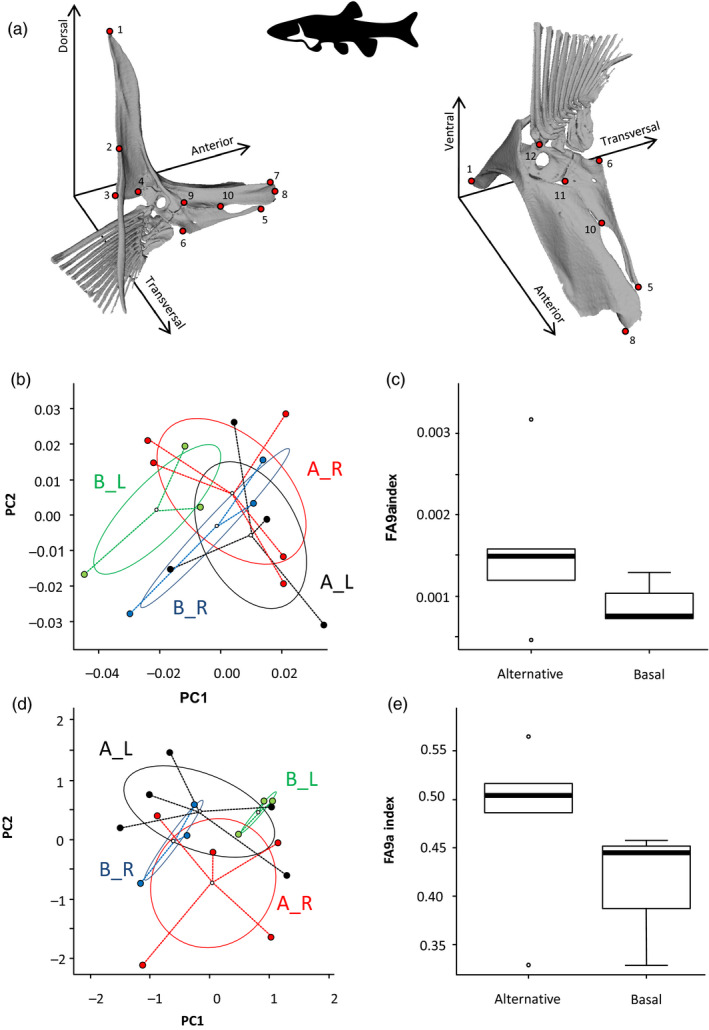
Fluctuating asymmetry in *Chrosomus eos‐neogaeus* clonal individuals. (a) Landmark scheme for the 3D analysis of the pectoral appendage. The landmarks were identified by red dots numbered from 1 to 12. Left and right images are two different points of view of the left pectoral appendage according to the fish orientation. (b‐e) Left–right variation was assessed through 3D morphometry of the pectoral appendage (b and c) and DNA methylation of lateral muscle (d and e). (b and d) Principal component analysis of the variation where groups are defined according to developmental pathway (B: basal; A: alternative) and side (L: left side; R: right side). (c and e) Box plot of FA9a index variation among individuals (Windig & Nylin, 2000)

Morphometric analyses performed on the pectoral appendage (Figure 2a) revealed a signal consistent with the one previously detected in the pharyngeal arches (Leung et al., 2017). Individuals from the alternative developmental pathway displayed lesser extent of directional asymmetry (Figure 2b; *R^2^*_alternative_ = 0.12 versus *R^2^*_basal_ = 0.22 for side effect on pectoral appendage shape variation) but higher fluctuating asymmetry assessed with FA9a index (Windig & Nylin, 2000) than these from the basal pathways (Figure 2c).

Likewise, preliminary results on 210 replicable loci (16.1% polymorphic) revealed similar variation in terms of DNA methylation profiles between left and right sides of individuals from the two developmental pathways. Individuals from the alternative developmental pathway displayed lesser extent of directional asymmetry (Figure 2d; *R^2^*_alternative_ = 0.13 versus *R^2^*_basal_ = 0.23 for side effects on DNA methylation profiles) but a trend for a higher fluctuating asymmetry (Figure 2e) than individuals from the basal developmental pathway. It is worth mentioning that low levels of variation are expected, considering the lack of genetic variation of these clonal hybrids. Nonetheless, while these preliminary results must be treated with caution due to the low sample size, epigenetic variation showed the same trend as morphological analyses. As expected, both revealed the higher fluctuating asymmetry in the developmental pathway leading to the alternative phenotype, while individuals from the basal developmental pathway displayed a clear directional asymmetry with less within‐group variation, suggesting greater stability of their development.

## 4 MICROBIOME

Throughout this paper, we described how the environment affects the phenotype of individuals. The environment encompasses abiotic (physicochemical conditions) and biotic (living organisms) factors. But where does the microbiome fit in this framework? The microbiome is composed of all the communities of microorganisms (bacteria, archaea, fungi, protists) that use another organism as habitat and can be conceptualized in different ways. At first glance, the host‐associated microbiome can be viewed as part of “the environment.” Its composition and metabolic activity represent a collection of extrinsic signals that influence the host at the physiological, ontological, and population scale.

However, this perspective undermines the symbiotic nature of the association members of these microbial communities form with their host, and with each other. The microbial cells living in and on an individual host sometimes exceed in number these of the host itself and always profoundly affect all aspects of its biology. These microbes provide nutrients and act as a primary defense against pathogens (Bennett, Alers‐Garcia, & Bever, 2006; Rosenberg, Koren, Reshef, Efrony, & Zilber‐Rosenberg, 2007). In animals, they are also involved in the development of the adaptive immune system (Sommer & Bäckhed, 2013), they mediate fat storage (Bäckhed et al., 2004), olfactory communication (Carthey, Gillings, & Blumstein, 2018), and can directly affect host behavior by interacting with the pathways for the synthesis (and degradation) of neurotransmitters (Stilling, Dinan, & Cryan, 2016).

This deep integration has led many biologists and philosophers to suggest the holobiont (the microbiome and its host) should be regarded as a single evolutionary individual (Bordenstein & Theis, 2015). From this perspective, the hologenome represents the genomic content of all organisms within the holobiont and its phenotype results from the integrated processes of all these components. Studies showing concordance between host phylogenies and microbiome assemblages (Brooks, Kohl, Brucker, van Opstal, & Bordenstein, 2016) provided evidence that the microbiome composition is directly influenced by the host's genotype. Even when reared in the same controlled environment, two hosts with different genetic backgrounds will develop different microbiota (Brucker & Bordenstein, 2012; Li, Zhu, Yan, Ringø, & Yang, 2014; Müller, Vogel, Bai, & Vorholt, 2016). This implies that during the early stage of life, the host participates in the co‐construction of the ecological niche of its symbionts (Borges, 2017; Cortese, Lu, Yu, Ruden, & Claud, 2016; Koleva, Kim, Scott, & Kozyrskyj, 2015), and in this sense, host–microbe interactions are more similar to reciprocal tissue–tissue signaling than to host–environment interactions.

Critics of this view argue that the lack of strict inheritance of the symbionts keeps the holobiont from being an individual in its evolutionary sense (Douglas & Werren, 2016; Skillings, 2016), and while we think that within the context of the new evolutionary synthesis the line between organisms and ecosystems needs to be redefined (Bourrat & Griffiths, 2018; Doolittle & Booth, 2017), we agree that given the definition of a Darwinian individual as a unit of selection (Godfrey‐Smith, 2013), the microbiota is better conceptualized as an epigenetic component of the holobiont than a part of its genetic repertoire.

Contrary to genetic information, the microbiome is not strictly hereditary (Opstal & Bordenstein, 2015). Instead, like the host epigenome which is established during the development by integration of genetic, environmental, and stochastic influences, the composition of the microbiome results from the integration of host, environmental, and stochastic factors (Gilbert et al., 2010; Li et al., 2014). Indeed, the microbiome is reconstructed de novo at each generation from (a) a “seed” microbiota that is directly transferred from parent to offspring (Koleva et al., 2015; Salem, Florez, Gerardo, & Kaltenpoth, 2015), (b) microbial partners acquired from the environment in a deterministic way by host–microbiota niche co‐construction (Bakke, Coward, Andersen, & Vadstein, 2015; Borges, 2017)—like epigenetic marks resulting from the genome‐encoded developmental program—and (c) other microbes according to the environmental context, history of colonization, and stochastic events (Carrier & Reitzel, 2017; Dethlefsen, Eckburg, Bik, & Relman, 2006). Once established, the host‐associated microbial communities are generally stable and resilient to external stressors. The core of both skin and gut microbial assemblages, for example, is maintained for years in healthy humans despite constant perturbations (Faith et al., 2013; Oh, Byrd, Park, Kong, & Segre, 2016). Yet, the microbiome is also dynamic and can shift from one stable composition to another following an alteration of the milieu (Quercia et al., 2014).

Because the microbiome affects the host phenotype without changing its DNA, it represents an epigenetic level of information between the host genotype and its phenotype (Stilling, Dinan, & Cryan, 2014). Furthermore, the microbiome is an epigenetic effector because some microbial metabolites literally are trans‐kingdom epigenetic vectors. Indeed, some noncoding RNAs (ncRNAs) produced by host‐associated bacteria can directly alter gene expression in eukaryotes. For example, Liu et al. (2012) showed that upon uptake by *C. elegans*, two ncRNA produced by *E. coli*, OxyS and DsrA, promoted (via RNA interference) the degradation of messenger RNAs from a chemosensory and a longevity‐associated gene, respectively. In assays measuring the grazing pressure of *C. elegans* on *E. coli*, this epigenetic manipulation of the host phenotype was beneficial to the bacteria. *C. elegans* can easily absorb environmental RNA, but such extra‐specific epigenetic control might be widespread as it appears that functional noncoding RNAs can be transferred from cell to cell across kingdoms through extracellular vesicles (Tsatsaronis, Franch‐Arroyo, Resch, & Charpentier, 2018). Assessing whether transduced epigenetic messengers are a common means of communication between hosts and their associated bacteria will require further investigation, but it is in our view an exciting new area of research.

So, what would considering the microbiome as an epigenetic component of the holobiont achieve? The conceptual framework that researchers adopt ahead of their studies dictates what the appropriate research questions would be (Lloyd, 2015). All three conceptions of the microbiome are valid in their own rights and there certainly are questions that cannot be addressed under our proposed framework (e.g., how is the host affected by interspecific competition within its microbiota?). Still, we think that considering the microbiome as an epigenetic level of host control would open way for new lines of research inquiries and hypotheses which would, unlike these brought up by considering the microbiome as part of the environment or as part of the holobiont genetic repertoire, place greater emphasis on microbiome variation and dynamism.

Considering the microbiome as an epigenetic trait of the holobiont, future studies may ask how various selection pressures shape the plasticity of the microbiome and in turn affect host fitness. We can expect for example that the microbiome composition and stability (e.g., the relative abundance of core versus facultative, and permanent versus transient partners) would result from ecological strategies such as environmentally‐driven plasticity and diversified bet‐hedging. This hypothesis predicts that a holobiont population adapted to an environment marked by unpredictable variations is likely to possess a microbiota characterized by more facultative and transient partners at the cost of greater vulnerability to pathogens.

## 5 DISCUSSION

In this paper, we proposed three lines of research centered on epigenetic variation in natural populations. Epigenetic processes enable the realization of different phenotypes from a given genotype, in response to environmental cues and through processing errors in the epigenotype. This appears to be a fundamental property of organisms to rapidly respond to temporal and spatial changes in environmental conditions without relying on genetic variation. It is especially relevant in the applied context of conservation that is expected to promote the resilience and long‐term self‐persistence of natural systems against anthropogenic environmental changes. If ecological strategies to face environmental fluctuations, developmental stability, and microbiomes are of paramount importance in ecology and evolution, this is particularly crucial in the context of global climate change that strongly modifies the nature, the magnitude, and the predictability of environmental changes.

In this context, the use of genetic tools has been proved essential to assess variation recorded during the long‐term history of populations and to define evolutionary significant units (ESUs; Ryder, 1986). These components are expected to represent the evolutionary potential of organisms to respond to environmental change. However, genetic variation is just one side of the medal. Despite the growing literature reporting genetic variation responsible for local adaptation to environmental conditions, little is known on how organisms deal with environmental fluctuations in terms of ecological strategies (Taudt, Colomé‐Tatché, & Johannes, 2016) and the genetic components of only a few plastic traits have been identified in model organisms (Gage et al., 2017; Mangin et al., 2017).

The pattern of epigenetic variation is expected to reflect the current biotic and abiotic conditions (e.g., Mirbahai & Chipman, 2014) as well as long‐term fluctuations, in terms of plasticity or diversified bet‐hedging adaptations, experienced by populations (Leung et al., 2016). Dispersal is also a crucial process to face environmental changes, and persistence in different environments can be facilitated by epigenetically induced mechanisms (Davidson, Jennions, & Nicotra, 2011; Kreß, Oppold, Kuch, Oehlmann, & Müller, 2017; Vogt, 2017). Epigenetic variation is then proposed to complete the whole picture of conservation biology (Eizaguirre & Baltazar‐Soares, 2014; Rey et al., 2019). A quantitative analysis of epigenetic variation to assess how the epigenetic marks have been established (i.e., randomly, genetically, or environmentally induced) brings additional precision to assess the relative role of phenotypic plasticity and diversified bet‐hedging used by organisms in a given environment (Leung et al., 2016).

Another challenge in conservation biology is to assess the damaging effects of environmental stresses on populations. Stressful conditions are expected to increase the probability of accidents during the development of individuals. This could alter the fitness and the risk of extinction of the population. Developmental stability was suggested as a surrogate to assess fitness in conservation biology (Clarke, 1995). Fluctuating asymmetry is recognized as an indicator of developmental stability (Dongen, 2006; Palmer & Strobeck, 2003). The use of epigenetic fluctuating asymmetry through a whole‐genome approach appears then as a relevant application in conservation biology.

Several papers (Redford, Segre, Salafsky, Rio, & McAloose, 2012; Trevelline, Fontaine, Hartup, & Kohl, 2019) highlighted the importance of considering microbiota in conservation, in the context of anthropogenic habitat disturbances. Environmental changes can influence the host–microbe symbiotic associations by altering microbial functions or community composition. Such disruptions may represent a serious threat to organisms. Hence, identifying the evolutionary forces that shape the resilience and dynamism of microbiomes becomes a necessity to assess how organisms will fair in the face of environmental disturbances. To this end, conceptualizing the microbiome as an acquired epigenetic component of the host emphasizes the role of host genotype, environment, and stochasticity in its establishment and highlights it as a source of variation which can be selected for by natural selection (Gilbert et al., 2010). Specifically, this framework allows plasticity and bet‐hedging strategies to be considered at the level of microbiome.

Numerous species do not exclusively lie on genetic variation to cope with environmental heterogeneity. Epigenetic processes allow for the production of different phenotypes without resorting to genetic variation. Such capacity is extremely valuable for genetically depauperate populations/organisms (Angers et al., 2010). However, epigenetic processes through phenotypic plasticity and diversified bet‐hedging can also be associated with the potential for population evolvability (Ashander, Chevin, & Baskett, 2016; De Jong, 2005; Gavrilets & Scheiner, 1993b; Ghalambor, McKay, Carroll, & Reznick, 2007; Price et al., 2003; Scheiner & Goodnight, 1984). They could slow down adaptive evolution by weakening the strength of selection if a given genotype displaying a broad range of high‐fitness phenotypes across environmental conditions. This could then mask genetic variation that would be otherwise disadvantageous when expressed (Pfennig et al., 2010; Price et al., 2003). Because selection pressures on natural populations could be largely altered by the different human activities (Hoffmann & Sgró, 2011), this hidden genetic variation could be advantageous in such conditions and help the population to recover from environmental pressure through these genetic changes (evolutionary rescue).

These changes at the level of intraspecific variation are not restricted to populations and species but are expected to have consequences at the level of the whole ecosystem. This paralleled the problem of scales in ecology: How do processes acting at the individual level scale up to communities (Levin, 1992)? The epigenotype is sensitive to the environment, and its effects on phenotype of individuals can affect ecological and evolutionary processes at the ecosystem level. For instance, populations of *Arabidopsis thaliana* harboring higher epigenetic variation display higher productivity as well as higher pest and competitor resistance (Latzel et al., 2013). Moreover, the contribution of phenotypic plasticity appeared the most important predictor in determining the species richness of plant communities (Barbour et al., 2019; Pérez‐Ramos, Matías, Gómez‐Aparicio, & Godoy, 2019). As the driver of the phenotypic plasticity, the epigenetic variation could then be considered as a relevant proxy of high levels of biodiversity.

Analysis of epigenetic variation has been proved useful in a broad range of studies related to the realization of phenotype. Its application could also be extended to several other fields traditionally investigated through genetic or phenotypic variation. Analyzed as a character, partition of epigenetic variation in a quantitative context could represent a promising molecular tool to assess the evolutionary potential of populations, in addition to the genotype, which represents a crucial step to better understand and conserve biodiversity. The relevance of the use of epigenetic variation could also be facilitated by recent technologies of sequencing enabling to assess both genetic and DNA methylation patterns. In conclusion, the epigenotype provides a highly valuable toolbox for the examination of the multiple phenomena affecting phenotypic outputs. It might be hoped that epigenetics, at the interface of several fundamental disciplines of biology, could stimulate collaboration in this crucial domain.

## CONFLICT OF INTEREST

None declared.

## Supporting information

Appendix S1

## Data Availability

Preliminary results presented in this manuscript are available from the corresponding author on request.
