# Supplementary material for: Sources of epigenetic variation and their applications in natural populations
Source: Evol Appl. 2020 Mar 18;13(6):1262–78. doi: 10.1111/eva.12946 (PMC7359850; doi:10.1111/eva.12946)
Supplement: Supplementary file 1 — Appendix S1 [file EVA-13-1262-s001.docx]

**SUPPLEMENTARY MATERIAL: Methods for asymmetry analyses**

Clonal hybrids *Chrosomus eos-neogaeus* were collected from a single lake (N 45° 57' 58.3'' W 74° 01' 43.5'') in the southern Quebec, Canada (Leung et al., 2017). Each individual was defined as symmetrical (1, 5 - 5, 1) or asymmetrical (1, 5 - 4, 1), according to the observed dental formulae of the 7th pharyngeal arch, using 3D computational images obtained by micro-CT scan (Leung et al., 2017). Eight individuals (3 and 5 individuals from basal and alternative developmental pathway, respectively) were analyzed in this preliminary study.

Geometric morphometric analyses of 3D models were used to measure the shape of the pectoral appendage. 3D models have been reconstructed from micro-CT scan according to Leung et al. (2017). Twelve landmarks were used to define the shape of both the left and right sides of the pectoral appendage (Fig 5A) and subject to a generalized procruste analysis using Geomorph package (Adams & Otárola‐Castillo 2013). We assessed landmark positioning error by placing landmarks twice for all individuals. Procruste’s analysis of variance (Klingenberg et al., 2002; Mardia, 2000) showed no landmark positioning session effect on the measured shape variation (*P* = 0.845).

Muscles from both sides of the body were removed for DNA extraction. Methylation of the DNA was accessed using the methylation-sensitive amplified polymorphism (MSAP) technique (Xiong et al., 1999) optimized by Leung et al. (2016), that allows the detection of cytosine methylation in amplified products by replicable PCR reactions. The obtained loci were used to construct a DNA methylation presence/absence matrix that we used as the epigenetic response variable for the following multivariate analyses

We assessed the level of directional asymmetry for the two developmental pathways, by quantifying the variation among sides using Procrustes ANOVA (Klingenberg et al., 2002; Mardia, 2000) or redundancy analyses (RDA; Borcard, Legendre, & Drapeau, 1992) for morphometrics and DNA methylation data respectively. Then, we assessed the level of fluctuating asymmetry for each individual by calculating the FA9a index (Windig & Nylin, 2000), *i.e.* one *minus* the correlation coefficient between the right and left (1 − *r*_R,L_) for each of the datasets. No statistical test was performed due to the low sample size in each group of developmental pathways.

**References:**

Adams, D. C., & Otárola‐Castillo, E. (2013). geomorph: an R package for the collection and analysis of geometric morphometric shape data. *Methods in Ecology and Evolution*, 4(4), 393-399. https://doi.org/10.1111/2041-210X.12035

Borcard D, Legendre P, Drapeau P. Partialling out the spatial component of ecological variation. *Ecology,* *1992*(73), 1045–1055. https://doi.org/10.2307/1940179

Klingenberg, C. P., Barluenga, M., & Meyer, A. (2002). Shape analysis of symmetric structures: Quantifying variation among individuals and asymmetry. *Evolution*, *56*(10), 1909–1920. https://doi.org/10.1111/j.0014-3820.2002.tb001 17.x

Mardia, K. (2000). Statistical assessment of bilateral symmetry of shapes. *Biometrika*, *87*(2), 285–300. https://doi.org/10.1093/biomet/87.2.285

Leung, C., Breton, S., & Angers, B. (2016). Facing environmental predictability with different sources of epigenetic variation. *Ecology and Evolution*, *6*(15), 5234–5245. <https://doi.org/10.1002/ece3.2283>

Leung, C., Duclos, K. K., Grünbaum, T., Cloutier, R., & Angers, B. (2017). Asymmetry in dentition and shape of pharyngeal arches in the clonal fish Chrosomus eos-neogaeus: Phenotypic plasticity and developmental instability. *PLoS ONE*, *12*(4), e0174235. https://doi.org/10.1371/journ al.pone.0174235

Windig, J. J., & Nylin, S. (2000). How to compare fluctuating asymmetry of different traits. *Journal of Evolutionary Biology*, *13*(1), 29–37. <https://doi.org/10.1046/j.1420-9101.2000.00143.x>

Xiong, L. Z., Xu, C. G., Maroof, M. A. S., & Zhang, Q. (1999). Patterns of cytosine methylation in an elite rice hybrid and its parental lines, detected by a methylation-sensitive amplification polymorphism technique. *Molecular and General Genetics*, *261*(3), 439–446. https://doi.org/10.1007/s0043 80050986
